# Supplementary material for: Small intestinal microbial dysbiosis underlies symptoms associated with functional gastrointestinal disorders
Source: Nat Commun. 2019 May 1;10:2012. doi: 10.1038/s41467-019-09964-7 (PMC6494866; doi:10.1038/s41467-019-09964-7)
Supplement: Supplementary file 1 — Supplementary Information [file 41467_2019_9964_MOESM1_ESM.pdf]

Small intestinal microbial dysbiosis underlies symptoms associated with functional gastrointestinal disorders

Saffouri, Shields-Cutler et al.

Supplementary Figures and Tables

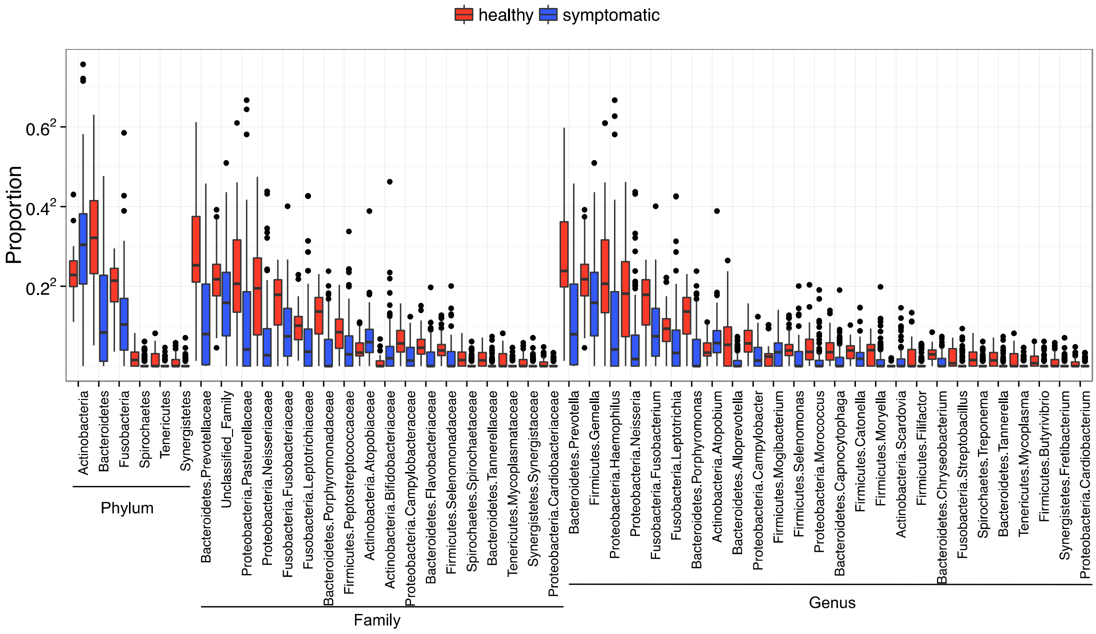

Supplementary Figure 1: Small intestinal microbial composition is significantly altered in symptomatic patients. Differences in relative abundances of microbial taxa at phylum, family, and genus levels among symptomatic patients and healthy controls (all FDR $q < 0.05$).

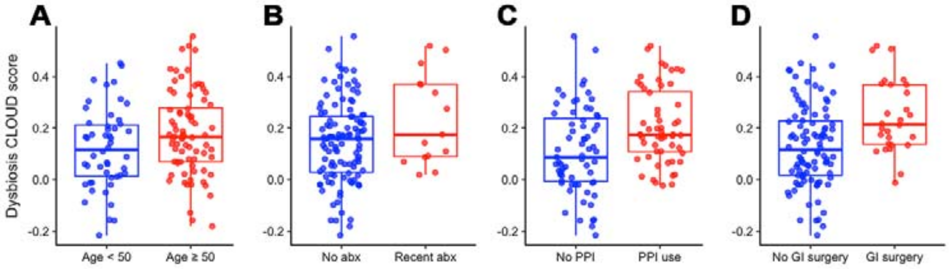

Supplementary Figure 2: Host characteristics influence DI score. Differences in DI score (symptomatic patients and healthy controls) based on **(A)** age, **(B)** antibiotic use, **(C)** PPI use, and **(D)** history of gastrointestinal surgery.

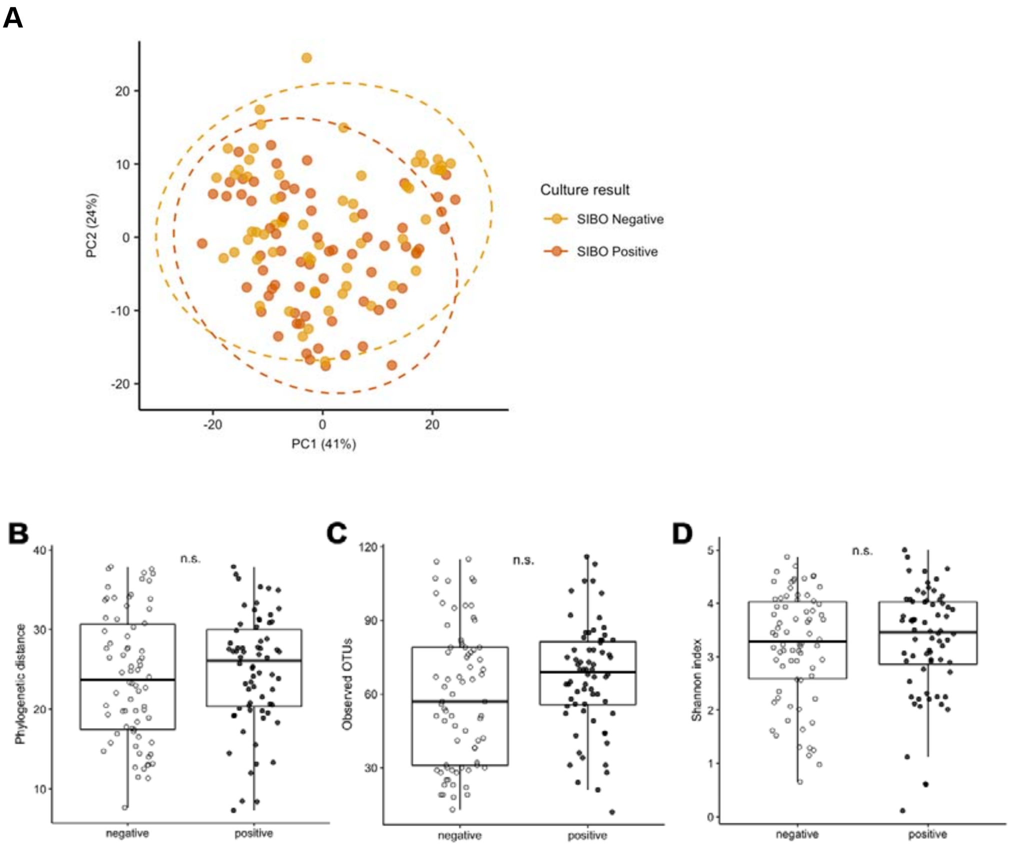

Supplementary Figure 3: Microbial diversity is not significantly different among patients with and without SIBO. (A) PCoA plot showing beta diversity of patients with GI symptoms (n=126) based on Aitchison distance; ellipses represent 95% confidence intervals. Alpha diversity of symptomatic patients that tested SIBO positive (n=66) or negative (n=60) based on **(B)** phylogenetic distance, (**C**) observed OTUs, and **(D)** Shannon metric (all $p > 0.05$, *t*-test; rarefied to 5,000 sequences).

A B

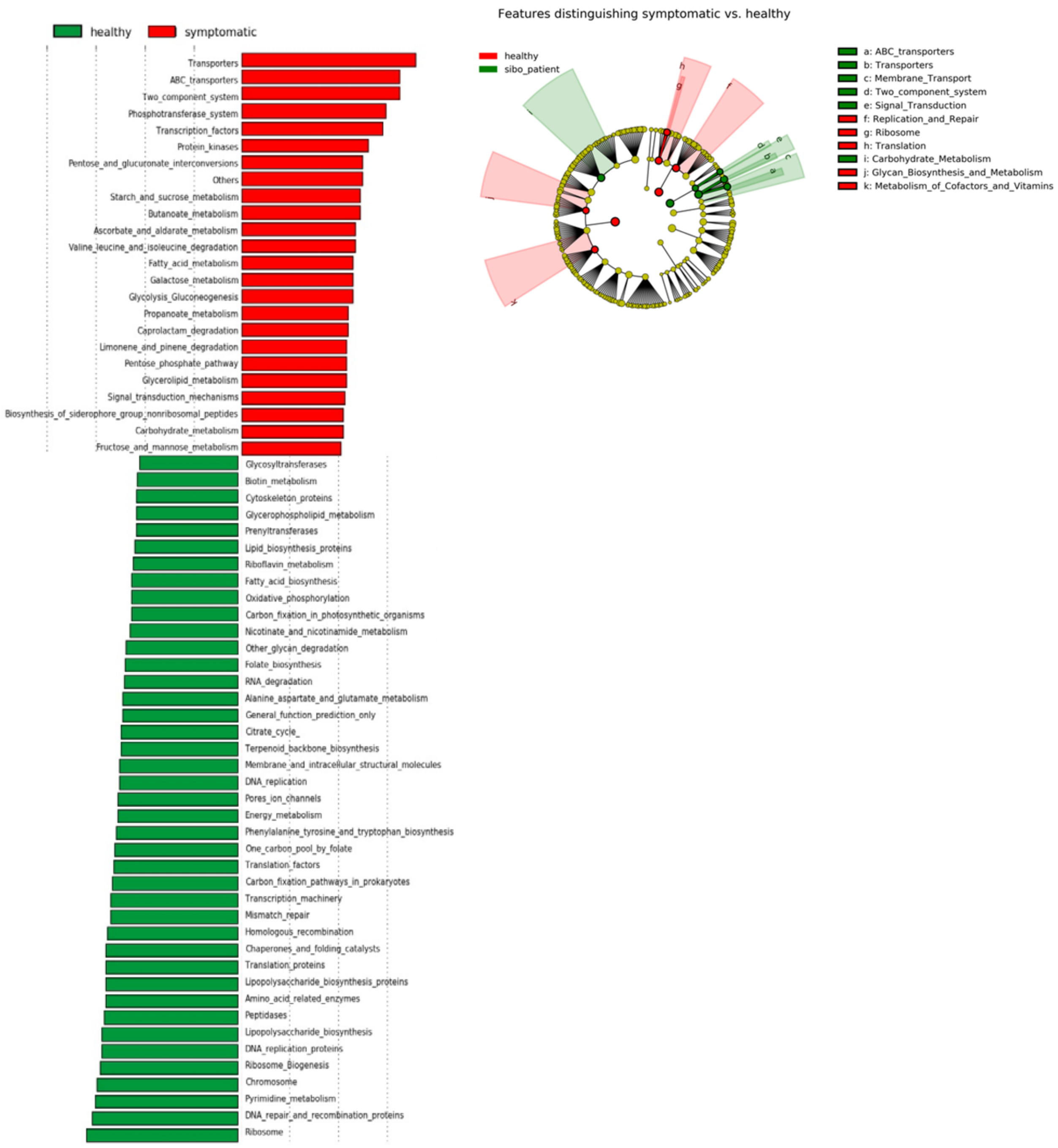

Supplementary Figure 4: Complex carbohydrate degradation pathways are enriched in healthy individuals. Differentially abundant microbial functions (LEfSe, LDA > 2) at the gene function level (L3; **A**) and pathway level (L2; **B**) imputed from microbial composition in patients with GI symptoms (n=126) and healthy controls (n=38).

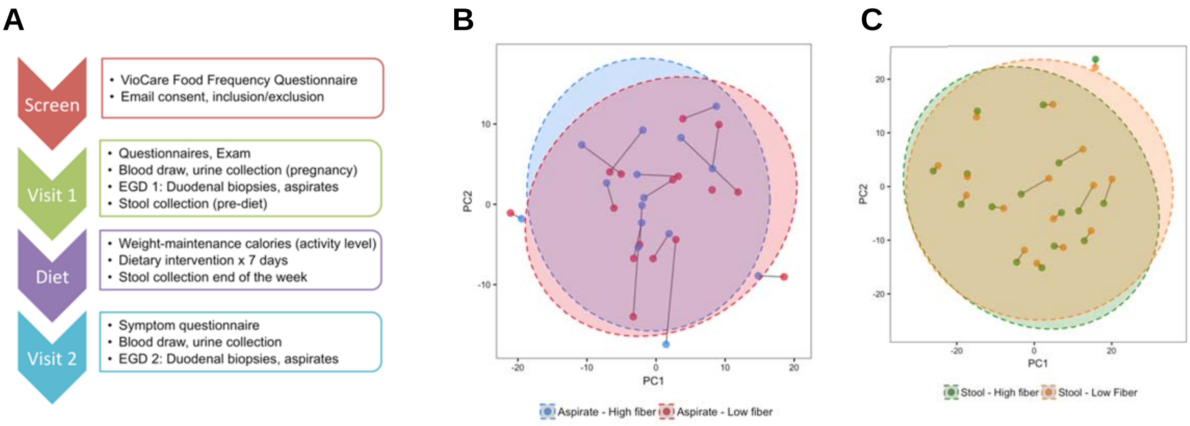

Supplementary Figure 5: Dietary intervention impacts stool and small intestinal microbiota. (A) Study design and dietary intervention in healthy individuals consuming high fiber diet. **(B, C)** Correlation between the within-individual microbial community composition before and after intervention in duodenal aspirate (**B**) and stool (**C**) by Procrustes analysis (p = 0.001, Monte Carlo simulation with 999 permutations).

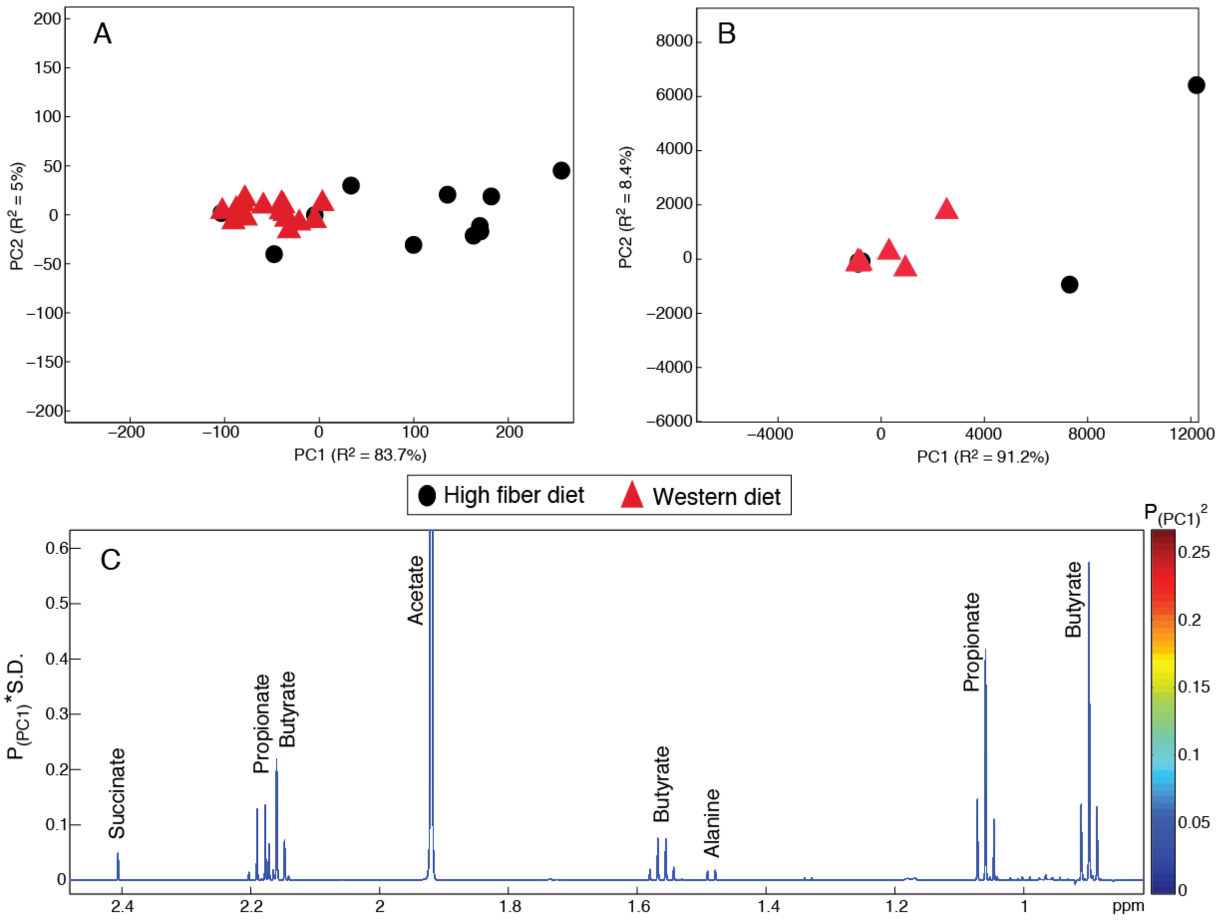

Supplementary Figure 6: **Dietary intervention impacts the metabolic profiles of fecal and duodenal samples.** Cross-validated scores plots from the PCA models built on the (**A**) fecal (R^2 = 88.7%) and (**B**) duodenal samples (R^2 = 99.6%). Back-scaled loadings plot (**C**) for principal component 1 (PC1) from the PCA model comparing pre- and post-intervention fecal samples.

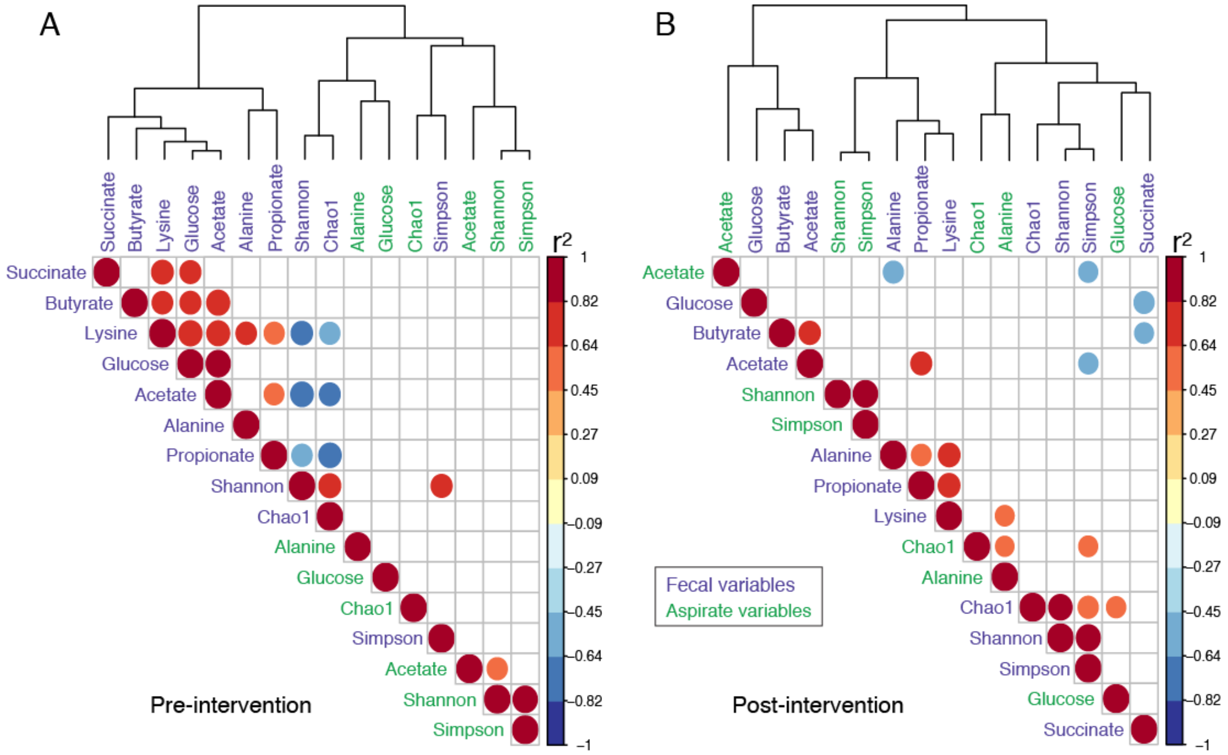

Supplementary Figure 7: Microbial diversity is associated with individual microbial metabolites. Individual correlodendograms showing statistical associations between fecal and duodenal metabolites and measures of alpha diversity in the duodenal and fecal microbiota (**A**) pre-intervention and (**B**) post-intervention. Color intensity and size of the circle are proportional to the correlation coefficients (Spearman correlation). Significant correlations ($p < 0.05$) are shown.

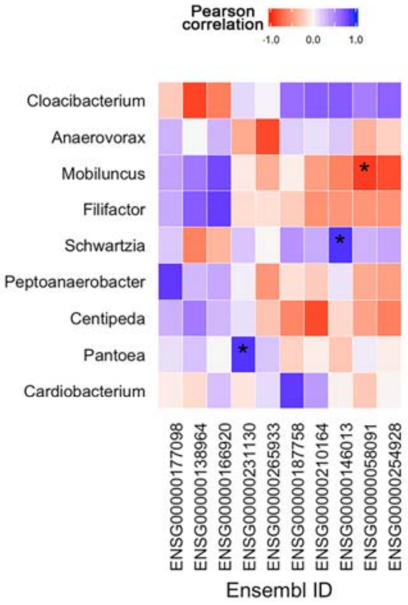

Supplementary Figure 8: Changes in the duodenal aspirate microbiota correlate with mucosal gene expression. Correlation of the log fold change in duodenal gene expression following dietary intervention with the change in CLR abundance of genus-level taxa (shown are correlations with FDR-adjusted *q*-value < 0.5; * indicates FDR-adjusted *q* < 0.25).

Supplementary Table 1: Contingency table showing similar dysbiosis classifications using the CLOUD method compared to spectral clustering.

		CLOUD	
		dysbiotic	healthy-like
spectral clustering	cluster 1	9	119
	cluster 2	29	7

Supplementary Table 2: Demographics of study participants

	**Participants (n=16)**
Mean age (years)	26
Sex (female %)	53.3
Race (Caucasian %)	73
Mean BMI (kg/m^2)	23.7
Duodenal bacterial growth >100,000 CFU/mL (positive %)	46.7

Supplementary Table 3: GI symptoms following dietary intervention

**Symptom**	**Affected n (%)**
Any	15 (100)
Any GI	12 (80)
Multiple (≥ 2)	14 (93)

Stool change (freq/consist)	11 (73)
Constipation (Rome III)	6 (40)
Fatigue /↓Energy	9 (60)
Only Fatigue/↓Energy	3 (20)
