# Supplementary material for: Small intestinal microbial dysbiosis underlies symptoms associated with functional gastrointestinal disorders
Source: Nat Commun. 2019 May 1;10:2012. doi: 10.1038/s41467-019-09964-7 (PMC6494866; doi:10.1038/s41467-019-09964-7)
Supplement: Supplementary file 3 — Description of Additional Supplementary Files [file 41467_2019_9964_MOESM3_ESM.pdf]

Description of Additional Supplementary Files

File Name: Supplementary Data 1
Description: Demographics and clinical features of symptomatic patients and healthy controls

File Name: Supplementary Data 2
Description: Taxonomic differences among small intestinal microbial communities from symptomatic patients and healthy volunteers

File Name: Supplementary Data 3
Description: Raw data for Figure 6A-C
