# Supplementary material for: Small intestinal microbial dysbiosis underlies symptoms associated with functional gastrointestinal disorders
Source: Nat Commun. 2019 May 1;10:2012. doi: 10.1038/s41467-019-09964-7 (PMC6494866; doi:10.1038/s41467-019-09964-7)
Supplement: Supplementary file 7 — Reporting Summary [file 41467_2019_9964_MOESM7_ESM.pdf]

nature research

Corresponding author(s): Purna Kashyap

Reporting Summary

Nature Research wishes to improve the reproducibility of the work that we publish. This form provides structure for consistency and transparency in reporting. For further information on Nature Research policies, see Authors & Referees and the Editorial Policy Checklist.

Statistical parameters

When statistical analyses are reported, confirm that the following items are present in the relevant location (e.g. figure legend, table legend, main text, or Methods section).

n/a	Confirmed	
☐	☒	The exact sample size (*n*) for each experimental group/condition, given as a discrete number and unit of measurement
☐	☒	An indication of whether measurements were taken from distinct samples or whether the same sample was measured repeatedly
☐	☒	The statistical test(s) used AND whether they are one- or two-sided<br>*Only common tests should be described solely by name; describe more complex techniques in the Methods section.*
☐	☒	A description of all covariates tested
☐	☒	A description of any assumptions or corrections, such as tests of normality and adjustment for multiple comparisons
☐	☒	A full description of the statistics including central tendency (e.g. means) or other basic estimates (e.g. regression coefficient) AND variation (e.g. standard deviation) or associated estimates of uncertainty (e.g. confidence intervals)
☐	☒	For null hypothesis testing, the test statistic (e.g. *F*, *t*, *r*) with confidence intervals, effect sizes, degrees of freedom and *P* value noted<br>*Give P values as exact values whenever suitable.*
☒	☐	For Bayesian analysis, information on the choice of priors and Markov chain Monte Carlo settings
☒	☐	For hierarchical and complex designs, identification of the appropriate level for tests and full reporting of outcomes
☐	☒	Estimates of effect sizes (e.g. Cohen's *d*, Pearson's *r*), indicating how they were calculated
☐	☒	Clearly defined error bars<br>*State explicitly what error bars represent (e.g. SD, SE, CI)*

Our web collection on statistics for biologists may be useful.

Software and code

Policy information about availability of computer code

Data collection	Illumina DNA sequence data (from MiSeq instrument) was processed using SHI7 (v0.9.2; https://github.com/knights-lab/shi7) and aligned to a closed-reference database of RefSeq genomes using BURST (v0.99.4; https://github.com/knights-lab/BURST).
Data analysis	Microbiome data was analyzed using QIIME (v1.9.1), PICRUSt (v1.1.3), LEfSe (Galaxy v1.0), and R (v3.3.3 and 3.5.0), with the R packages vegan, ggplot2, randomForest, boruta, tidyverse, reshape2, and psych. Applications of these and published methods to generate indices reported in the results are further provided on GitHub (https://github.com/RRShieldsCutler/small_bowel_dysbiosis/). 1H-NMR data was processed in MATLAB Version 8.3.0.532 R2014a. RNAseq data was analyzed with the Mayo Analysis Pipeline for RNA Sequencing, and is described and cited in the Methods.

For manuscripts utilizing custom algorithms or software that are central to the research but not yet described in published literature, software must be made available to editors/reviewers upon request. We strongly encourage code deposition in a community repository (e.g. GitHub). See the Nature Research guidelines for submitting code & software for further information.

Data

Policy information about availability of data

All manuscripts must include a data availability statement. This statement should provide the following information, where applicable:

- Accession codes, unique identifiers, or web links for publicly available datasets
- A list of figures that have associated raw data
- A description of any restrictions on data availability

The 16S amplicon DNA sequences, metabolomics data, and RNAseq data have all been deposited to public databases; details have been included in the Data Availability section of the Methods. Additional raw data has been provided as Supplementary Data files along with the manuscript.

Field-specific reporting

Please select the best fit for your research. If you are not sure, read the appropriate sections before making your selection.

☒ Life sciences ☐ Behavioural & social sciences ☐ Ecological, evolutionary & environmental sciences

For a reference copy of the document with all sections, see nature.com/authors/policies/ReportingSummary-flat.pdf

Life sciences study design

All studies must disclose on these points even when the disclosure is negative.

Sample size	The first study on small bowel aspirates was an exploratory study and consecutive samples were collected from the laboratory. The diet study was a pilot study investigating the effect of dietary fiber.
Data exclusions	Sequence data was filtered to read quality >=32. For taxonomic analyses, removed singleton OTUs and those present in less than 1% of samples and dropped samples with <1000 reads.
Replication	All DNA extraction and sequencing was carried out using previously published protocols that have been rigorously evaluated for technical reproducibility. Based on previous findings we expect that technical variation will be smaller than biological variation in our sequencing data. in terms of the human studies, the findings will need to be confirmed in separate cohorts but that was not done as part of this study.
Randomization	There was no control arm as the patient served as their own control, hence there was no randomization
Blinding	This was an open label pilot study and both physician and volunteer were aware of the dietary intervention.

Reporting for specific materials, systems and methods

Materials & experimental systems

n/a	Involved in the study	
☒	☐	Unique biological materials
☒	☐	Antibodies
☒	☐	Eukaryotic cell lines
☒	☐	Palaeontology
☒	☐	Animals and other organisms
☐	☒	Human research participants

Methods

n/a	Involved in the study	
☒	☐	ChIP-seq
☒	☐	Flow cytometry
☒	☐	MRI-based neuroimaging

Human research participants

Policy information about studies involving human research participants

Population characteristics	We evaluated 126 symptomatic (21% male; age 15-89 years, median 55 years) patients who underwent EGD with duodenal aspirate collection; the major symptoms reported included diarrhea (45%), abdominal pain (28%), and bloating (13%). Of these patients, 66 (52%) tested positive for SIBO, while 60 patients (48%) tested negative for small intestinal bacterial overgrowth (SIBO). Among the 66 who tested positive, 49 were positive for anaerobic bacterial overgrowth, 17 were positive for mixed anaerobic and aerobic overgrowth and none were positive for aerobic bacterial overgrowth alone. All healthy individuals tested negative for SIBO.

Recruitment

Consecutive small bowel aspirate samples submitted following diagnostic endoscopy in symptomatic patients were obtained directly from the microbiology laboratory from May, 2016 through December, 2016. Duodenal aspirates were collected during esophagogastroduodenoscopy (EGD) following direct passage to the duodenum with minimal inflation in the stomach using a standard aspiration catheter passed through suction port during endoscopy. Samples associated with patients who previously provided consent allowing for review of electronic medical record were included. A total of 143 aspirates from symptomatic patients were obtained over this time interval. After excluding samples due to lack of consent or low read depth on sequencing a total of 126 symptomatic patients was included. Duodenal aspirates obtained from 38 healthy volunteers participating in other research studies and collected in a similar manner were also obtained and processed similarly. Clinical metadata was obtained by retrospective review of electronic medical record including demographic information, body mass index (BMI), clinical indication for small intestinal bowel overgrowth (SIBO) testing, quantitative aerobic and anaerobic aspirate culture results, antibiotic course for treatment of SIBO, clinical response to antibiotics, need for repeat antibiotics, recent medications including antibiotics and proton pump inhibitor use, gastrointestinal surgeries, comorbid conditions etc.

Dietary intervention study in healthy volunteers
This was a pilot prospective single-center dietary intervention study in healthy volunteers consuming a high fiber diet at baseline. The study was registered with clinicaltrials.gov NCT03266536.
Participants: Eligible subjects were healthy adults (≥18 years) with baseline fiber intake ≥11 g/1000 calories/day; <10% daily calories from added sugar; ≥5 servings of fruits and vegetables/day; and ≤13% daily calories from saturated fat based on completed food frequency questionnaire. Patients were excluded from the study if they did not meet the above diet requirements, had a known diagnosis of inflammatory bowel disease, microscopic colitis, celiac disease or other inflammatory conditions, presence of abdominal symptoms based on baseline questionnaire, oral antibiotic or probiotic use within the past 4 weeks, pregnancy or plans to become pregnant within the study time frame, or any other disease(s), condition(s) or habit(s) that would interfere with completion of study.
Screening and study procedures: All subjects were screened in person or by phone and completed a food frequency questionnaire to ensure they met inclusion and exclusion criteria. At the initial and follow up visit they underwent EGD with conscious sedation. Duodenal aspirates were obtained via a standard suction catheter passed through the suction port with minimal inflation in the stomach and an aliquot was submitted to clinical laboratory for testing for SIBO and the remaining was stored for microbiome analysis at-80. Eight duodenal biopsies were obtained for Ussing chamber studies, microbiome analysis and host RNA seq. At initial and follow up visits, participants completed a symptom and demographic questionnaire and provided a stool sample. Symptoms assessed on questionnaires included: stool frequency, straining, incomplete evacuation, hard/lumpy stools, abdominal pain associated with bowel movements, diarrhea/loose-watery stools, bloating, swallowing difficulties, nausea/vomiting, heartburn, fatigue, and appetite. Answers were recorded in a binary (yes/no) fashion. Intervention: All participants consumed a 7-day standardized diet with typical United States macronutrient calorie distribution: 50% from carbohydrates, 35% from fats, and 15% from protein. The diet was low in fiber (<10 g/1000 calories/day) and high in simple sugar (≥50% of daily carbohydrates). Dietary instructions and food choices were discussed at the index, pre-intervention visit by a licensed dietician.
